# Alteration of Fecal Microbiota, Fecal Metabolites, and Serum Metabolites in Dairy Cows with Pre-Retained Placenta

**DOI:** 10.3390/metabo14070386

**Published:** 2024-07-15

**Authors:** Tao Zhou, Zhenlong Du, Zhengzhong Luo, Xiaoping Li, Dan Wu, Yixin Huang, Kang Yong, Xueping Yao, Liuhong Shen, Shumin Yu, Zuoting Yan, Suizhong Cao

**Affiliations:** 1 Department of Clinical Veterinary Medicine, College of Veterinary Medicine, Sichuan Agricultural University, Chengdu 611130, China; 2 Lanzhou Institute of Animal Husbandry and Veterinary Pharmaceutical, Chinese Academy of Agricultural Sciences, Lanzhou 730050, China; 3 Department of Clinical Veterinary Medicine, College of Veterinary Medicine, China Agricultural University, Beijing 100000, China; 4 Department of Animal Husbandry & Veterinary Medicine, College of Animal Science and Technology, Chongqing Three Gorges Vocational College, Chongqing 404105, China

**Keywords:** dairy cows, retained placenta, early-warning markers, omics technology

## Abstract

Retained placenta (RP) affects lactation and fertility in dairy cows and causes economic losses to the dairy industry. Therefore, screening for early warning of this disease is important. This study used multi omics techniques to reveal the metabolic differences of dairy cows before RP onset and to find potential warning markers. Fecal samples and serum samples of 90 healthy Holstein cows were collected 7 days pre-calving; 10 healthy and 10 RP cows were enrolled according to normal expulsion of fetal membranes after calving. Fecal samples were subjected to 16S rRNA sequencing and untargeted metabolomics analysis, while plasma was analyzed using targeted metabolomics. Pathogenic bacteria levels increased in the intestines of cows with RP compared to those in healthy cows. Lipid metabolites constituted the largest proportion of differential metabolites between feces and plasma. Six potential warning markers for RP in cows were identified, including two fecal microbiomics markers (*Oscillospiraceae UCG-005* and *Escherichia-Shigella*), one fecal untargeted metabolomics marker (*N*-acetylmuramic acid), and three plasma targeted metabolomics markers (glycylcholic acid-3 sulfate, 7-ketolithocholic acid, and 12-ketolithocholic acid). These biomarkers can predict RP occurrence in the early perinatal period. These results lay a theoretical foundation for early nutritional intervention and pathogenesis research in dairy cows.

## 1. Introduction

Health problems in dairy cows affect milk yield and quality. Perinatal diseases in dairy cows have always been a critical factor hindering the development of the dairy industry and affecting dairy cow health [1]. Perinatal diseases mainly include a retained placenta (RP), ketosis, and abomasum displacement. RP is a common reproductive disease in dairy cows caused by multiple factors after calving [2]. The clinical symptom is the failure to discharge the fetal membranes within 8 h of calving, although some studies have defined this as within 12 h [3]. According to research statistics, the incidence of RP is approximately 10–30%, and up to 50% in summer [4]. RP is mainly related to farm management (drug abuse), environment (stress), the physiological state of dairy cows (age, parity, hormone level, genetic status, and nutritional factors), and the actual physiological status of the calf (stillbirth, multiple births, and dystocia) [5]. Cows with RP are prone to other secondary diseases, including endometritis, mastitis, and septicemia. This leads to a decrease in milk yield, shortening of lactation days, a decrease in reproductive performance, and even the passive elimination and death of dairy cows [6]. The economic loss caused by each case of RP is approximately USD 285 or GBP 239 [7]. Therefore, identifying early-warning biomarkers for RP in dairy cows has great economic value.

Multiple changes in hormones, the immune system, and metabolism occur in dairy cows before and after calving, which is a critical period for immune system development, metabolic balance, lactation initiation, and fertility [8,9]. A study showed that plasma concentrations of urea, triglyceride, β-hydroxybutyric acid, and non-esterified fatty acids increased during the perinatal period in RP cows, whereas blood sugar and total cholesterol decreased [10]. In terms of minerals, magnesium and phosphorus content increases during the week of illness, while the calcium content decreases. These indicators can all be used as early-warning indicators of RP in dairy cows in the early stage of the perinatal period [11]. Targeted metabolomics showed that cows undergo multiple changes in amino acid metabolism pathways before developing RP [12]. Cows with RP exhibit immune suppression and oxidative stress during the early stages of the perinatal period [13]. The balance of intestinal microflora and abnormal bile acid metabolism are closely related to the development of dairy cow diseases [14,15,16]; however, there is a lack of research on the metabolism of gut microbiota and plasma metabolites in cows with RP.

A significant feature of omics technology is its ability to reveal the overall characteristics of cells, tissues, or organisms under the influence of environmental factors [17]. Therefore, its utilization provides important clues to study changes during the perinatal period of dairy cows [18] and provides an important basis for studying the metabolic changes of cows during the perinatal period.

In our previous study, we used a multi-panel technique to analyze the correlation between fecal microbes and metabolites and plasma metabolites in healthy and abomasum-displaced dairy cows. The identification of metabolites (secondary bile acids and short-chain fatty acids) that are closely associated with dysbiosis of the gut microbiota confirmed the interaction between the host and the gut microbiota [15]. Additionally, the information provided by metabolomics is closer to the phenotype and better reflects the physiological characteristics of cows. Therefore, this study was conducted with the objective to use 16S rRNA gene amplicon sequencing and metabolomics for revealing metabolic changes in healthy cows and RP cows 7 days before calving from the perspective of body metabolism and to identify potential early-warning biomarkers of RP cows with good sensitivity, specificity, and stability to reduce the incidence of RP in dairy cows.

## 2. Materials and Methods

### 2.1. Animal Management and Sample Collection

The experimental protocol was approved by the Institutional Animal Care and Use Committee of Sichuan Agricultural University (No. DYY-2018203039, 28 March 2018). The experiment was conducted at an intensive farm with approximately 800 head of lactating dairy cows in Sichuan from January to June 2022. A total of 90 healthy perinatal primiparous cows of similar age and with similar body condition scores and expected dates of calving were selected. These cows were fed in a cowshed with 90 stalls. A total mixed ration diet was fed twice daily during the experiment. The dietary formula and dry matter intake are described in Supplementary Table S1. According to the expected calving record, feces and serum samples were collected from the rectum 7 days before expected calving dates. The serum samples were stored at −80 °C. Fecal samples were divided into sterile test tubes and stored in liquid nitrogen to analyze fecal microbiota and metabolites. Cattle with a normal discharge of the placenta after calving were used as controls. Cattle whose real calving date deviated from the expected calving date and abnormal cattle (premature delivery, dystocia, twins, mastitis, milk fever, Ketosis, and so on) were excluded, and 10 healthy (HE) cows and 10 cows with RP were randomly screened.

### 2.2. 16S rRNA Amplicon Sequencing Data Processing and Analysis

The fecal microbiome genome was extracted using the cetyltrimethylammonium bromide method, and the quality of the extracted DNA was determined using agarose gel electrophoresis (2%). The V3–V4 region of 16S rRNA was amplified by polymerase chain reaction using the universal primers 341F (5′-CCTACGGNGGCWGCAG-3′) and 805R (5′-GACTACHVGGGTATCTAATCC-3′). The amplified products were recovered and purified by 2% agarose gel electrophoresis. A NovaSeq 6000 sequencing platform(Kapa Biosciences, Woburn, MA, USA) was used for 2 × 250 bp paired-end sequencing with a NovaSeq 6000 SP Reagent Kit (Beckman Coulter Genomics, Danvers, MA, USA). The original data were spliced by reads, filtered by tags, and dechimerized to obtain valid data. Diversity analysis, species annotation, and difference analysis were performed based on the obtained Amplicon Sequence Variant (ASV) feature sequence and ASV abundance table.

### 2.3. Fecal Untargeted Metabolomic Analysis

Twenty fecal samples were slowly thawed at 4 °C, and an appropriate amount was added to a precooled methanol/acetonitrile/water solution (2:2:1, *v*/*v*), vortexed, mixed, subjected to low-temperature ultrasound for 30 min, kept at −20 °C for 10 min, and centrifuged at 14,000× *g* for 20 min at 4 °C. The supernatant was vacuum-dried. During mass spectrometry analysis, 100 µL of aqueous acetonitrile solution (1:1, *v*/*v* acetonitrile: water) was added for reconstitution, vortexed, centrifuged at 14,000× *g* for 15 min at 4 °C, and the supernatant was collected for analysis. Liquid chromatography–mass spectrometry (1290 Infinity LC, Agilent Technologies, Santa Clara, CA, USA) conditions and electrospray ionization/tandem mass spectrometry (AB SCIEX, Toronto, ON, Canada) conditions are described in detail in the Supplementary Materials.

### 2.4. Serum Metabolomic Analysis

After the samples were slowly thawed at 4 °C, 200 µL of plasma sample was mixed with 800 µL of precooled acetonitrile/methanol (1:1, *v*/*v*) solution containing five deuterated internal standards to compensate for ionization losses. All mixtures were sonicated for 30 min in an ice water bath, followed by incubation at −20 °C for 60 min and centrifugation at 14,000× *g* for 20 min at 4 °C. The supernatant was collected and vacuum-dried. A total of 100 µL acetonitrile/water (1:1, *v*/*v*) mixture was added, mixed, and centrifuged at 14,000× *g* for 15 min at 4 °C before ultraperformance liquid chromatography–tandem mass spectrometry (1290 Infinity LC; Agilent Technologies) analysis was performed. Chromatographic and mass spectrometry conditions are described in detail in the Supplementary Materials. Plasma samples were sent to Shanghai Applied Protein Technology Co., Ltd for targeted metabolomic mapping.

### 2.5. Statistical Analysis

R software (v 4.13) was used for univariate analyses between the HE and RP groups, including the Wilcoxon rank-sum test or Student’s *t*-tests and fold changes. The specific methods used for raw data processing and identification of fecal metabolites were consistent with those of our previous report [19]. The metabolomics data were subjected to *t*-tests and multivariate statistical analyses, including principal component analysis, partial least-squares discriminant analysis, and orthogonal partial least-squares discriminant analysis (OPLS-DA) to reflect the trend and difference in sample distribution between the HE and RP groups, and a permutation test was used to ensure the stability of the model. Variable importance in projection (VIP) > 1 and *p* < 0.05 were used as criteria for screening differential metabolites. The selected differential metabolites were analyzed by cluster analysis and metabolic pathway analysis using the MetaboAnalyst 5.0 online analysis platform. Linear discriminant analyses (LDA) > 2 and *p* < 0.05 were used as the standard for screening differential microbiota. Spearman’s correlation analysis of differential fecal microbiota and fecal metabolites was performed using an online tool (https://www.omicstudio.cn/tool, accessed on 22 March 2023). A receiver operating characteristic (ROC) curve was used to evaluate the predictive ability of the top-ranked potential markers for RP in dairy cows.

## 3. Results

### 3.1. Fecal Microbiome Alterations between the HE and RP Groups

**Figure 1:**
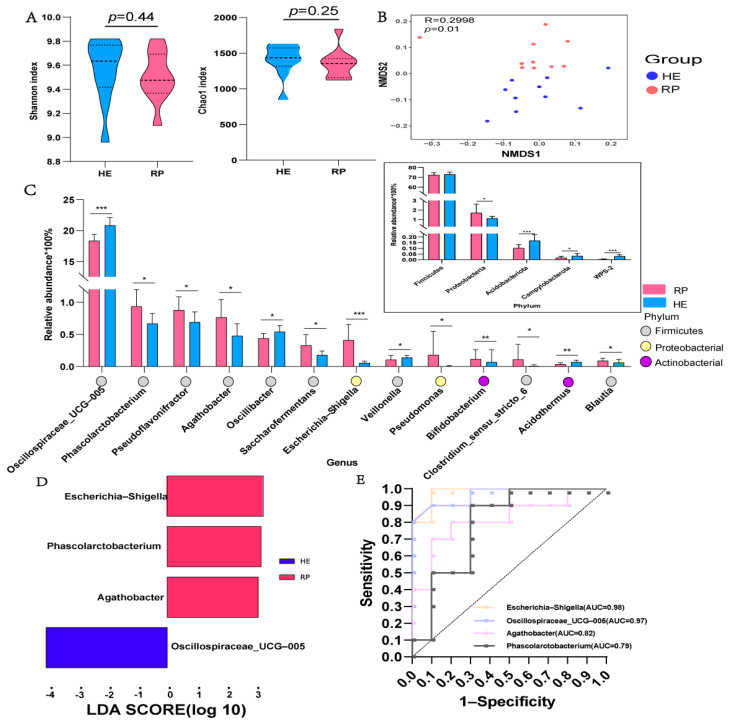
The fecal microbiome differs between the healthy (HE) and retained placenta (RP) groups. (**A**,**B**) Alpha and beta diversity of bacterial communities between the HE and RP groups. (**C**) The difference in fecal microbiota at the genus and phylum levels between the HE and RP groups (only representing the genera with relative abundance > 0.05), and the changes at the phylum level in the box. * *p* < 0.05, ** *p* < 0.01, *** *p* < 0.001. (**D**) Linear discriminant analysis of the differential bacteria between the HE and RP groups. (**E**) The receiver operating characteristic (ROC) curve of key fecal bacteria. AUC, area under the curve; LDA, least discriminant analysis.

After removing chimeras and low-quality sequences, 1,029,680 valid tag sequences were obtained, with an average of 51,484 high-quality gene sequences per sample for follow-up analysis. A total of 7576 ASVs were obtained. The two groups of samples shared 2797 ASVs, and 2554 and 2225 ASVs were identified in the HE and RP groups, respectively (Supplementary Material Figure S1). When the sequencing depth was 10,000, the sparse curve was stable, indicating that the sequencing quality was good and that it had a certain depth and representativeness (Supplementary Figure S2). Alpha-diversity analysis showed no significant differences in the Shannon and Chao1 indices between the HE and RP groups (*p* > 0.05) (Figure 1A). Beta-diversity analysis showed significant non-metric multidimensional scaling separation between the HE and RP groups (*p* < 0.05) (Figure 1B).

Firmicutes, Verrucomicrobia, Bacteroidota, and Proteobacteria were the main phyla in the HE and RP groups, accounting for over 95% (Supplementary Table S2). The abundance of Proteobacteria significantly increased in the RP group compared with that in the HE group (*p* < 0.05), whereas the abundance of Acidobacteriota, Campylobacterota, and WPS-2 significantly decreased in the RP group (*p* < 0.05) (Figure 1C). At the genus level, there were 56 differential genera between the HE and RP groups (*p* > 0.05). We mainly focused on differential genera with a relative abundance > 0.05. Among them, the abundances of *Phascolarctobacterium*, *Pseudoflavonifractor*, *Agathobacter*, *Saccharofermentans*, *Escherichia-Shigella*, *Pseudomonas*, *Bifidobacterium*, *Clostridium_sensu_stricto_6*, and *Blautia* significantly increased in the RP group (*p* < 0.05), and the abundances of *Oscillospiraceae_UCG-005*, *Oscillibacter*, *Veillonella*, and *Acidothermus* significantly decreased in the RP group (*p* < 0.05) (Supplementary Table S3). The LDA of *Escherichia-Shigella*, *Oscillospiraceae_UCG-005*, *Agathobacter*, and *Phascolarctobacterium* yielded a value > 2 (Figure 1D). *Oscillospiraceae_UCG-005* and *Escherichia-Shigella* were selected as potential early-warning biomarkers for RP by combining the results with the area under the ROC curve (Figure 1E).

### 3.2. Quality Control Analysis and Model Reliability Testing

A multivariate quality control chart was used to test all plasma and fecal samples to determine the reliability of the model. Fecal and serum samples did not exceed the area less than × 2 above the x-axis, and most of the test samples were near the control limit (Supplementary Figures S3 and S6). OPLS-DA was performed on plasma and fecal samples (Supplementary Figures S4 and S7 left), revealing a significant separation between the HE and RP groups. A permutation test was used to assess the model to avoid overfitting supervised models during modeling. The R2 and Q2 of the random model became lower as the displacement retention gradually decreased, indicating good model stability (Supplementary Figures S5 and S7 right).

### 3.3. Alterations in the Fecal Metabolic Profiles between the HE and RP Groups

**Figure 2:**
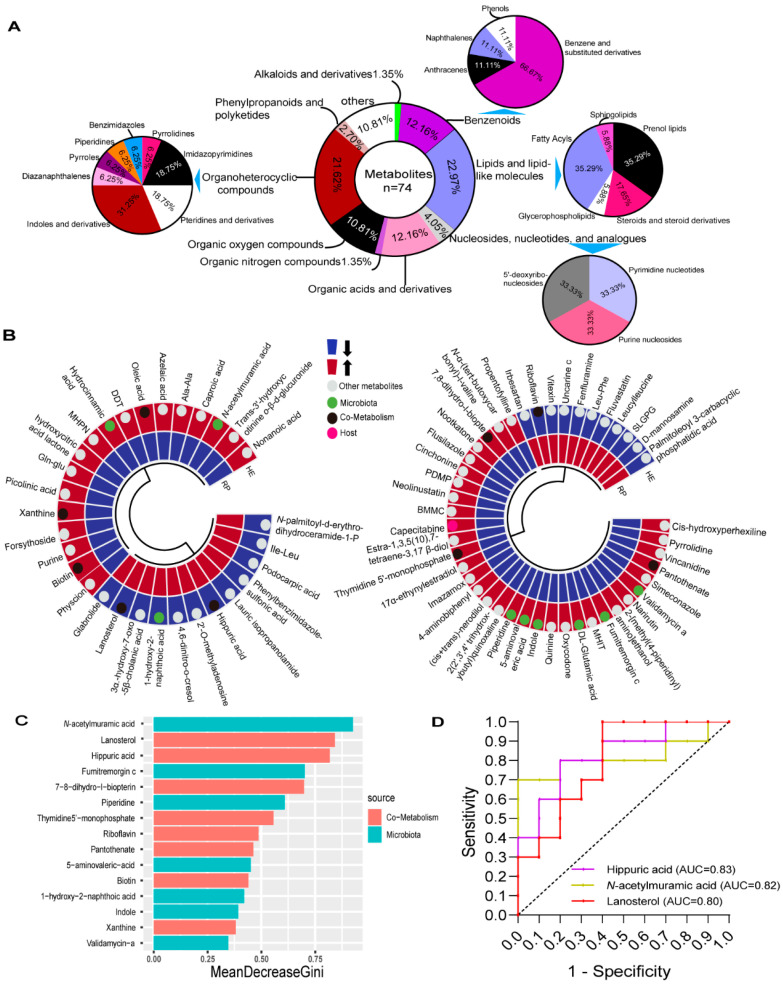
Differential fecal metabolites between the HE and RP groups. (**A**) The hollow pie chart represents the superclass ratio of 74 metabolites. The solid pie chart represents the proportion of metabolites in a single superclass. (**B**) Clustering heatmap of significantly different metabolites in cow feces between the HE and RP groups under positive (left) and negative (right) ion modes. (**C**) Random forest Gini index regression analysis ranks differential metabolites in descending order of importance of model accuracy by their relative abundance. (**D**) The ROC curve of differential fecal metabolites.

Differential metabolites were screened using VIP > 1 and *p* < 0.05 as the criteria (Supplementary Table S4). In total, 74 significant differential metabolites were identified between the HE and RP groups in the positive and negative ion modes. Using the HMDB database (https://hmdb.ca/, accessed on 25 June 2024), the 74 significant differential metabolites were classified. These metabolites were mainly organoheterocyclic compounds (21.62%), organic oxygen compounds (10.81%), lipids and lipid-like molecules (22.97%), nucleosides, nucleotides, and analogs (4.05%), benzenoids (12.16%), organic acids and derivatives (12.16%), others (10.81%), phenylpropanoids and polyketides (2.70%), alkaloids and derivatives (1.35%), and organic nitrogen compounds (1.35%) (Figure 2A). Heat maps showed that 41 differential metabolites were increased, while 33 were decreased in the feces of the HE group compared with those of the RP group (Figure 2B). The sources of these differential metabolites were determined using MetOrigin (http://metorigin.met-bioinformatics.cn/home/, accessed on 25 June 2024) (Figure 2B). These metabolites included one host metabolite, nine bacterial metabolites, nine host–bacterial co-metabolites, and 55 other metabolites. The random forest model ranked 19 valuable differential metabolites in descending order of importance, showing only the top 15 metabolites (Figure 2C). Subsequently, these metabolites were analyzed using the ROC curve (Supplementary Table S5). The area under the curve (AUC) of all metabolites was >0.75, and the AUC of *N*-acetylmuramic acid, lanosterol, and hippuric acid was >0.80 (Figure 2D). We selected N-acetylmuramic acid as a potential early-warning marker of RP in dairy cows based on the Gini index and AUC. 

### 3.4. Alterations in Serum Metabolic Profiles between the HE and RP Groups

**Figure 3:**
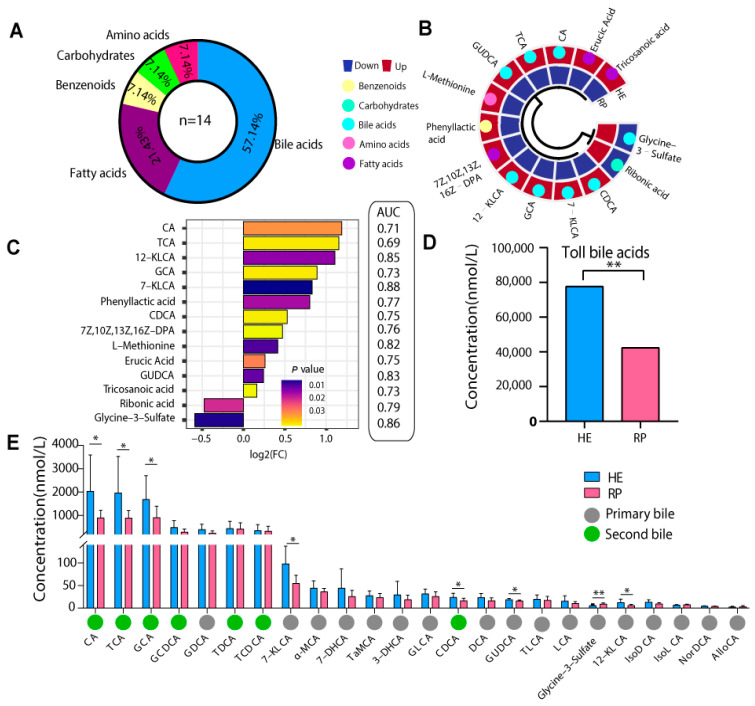
Differential serum metabolites between the HE and RP groups. (**A**) The hollow pie chart represents the class ratio of 14 differential metabolites. (**B**) The clustering heatmap of the significantly different metabolites in the plasma of the dairy cows between the HE and RP groups. (**C**) Histogram of log2FC and *p* value of differential bile acids between the HE and RP groups. (**D**) The bar chart represents the abundance of total bile acids in the HE group and RP group. (**E**) The bar chart represents the relative abundance of different bile acids in the plasma of cows in the HE group and RP group. Blue and pink represents the HE and RP groups, respectively. * *p* < 0.05, ** *p* < 0.01. CA, cholic acid; CDCA, chenodeoxycholic acid; GCA glycylcholic acid GUDCA, glycine ursodeoxycholic acid; 7-KLCA, 7-ketolithocholic acid; 12-KLCA, 12-ketolithocholic acid; TCA, taurocholic acid.

A total of 14 metabolites with significant differences were screened in the plasma of cows in the HE and RP groups (VIP > 1 and *p* < 0.05). Among them, bile acids (BAs) were 57.14%, fatty acids were 21.43%, and amino acids, carbohydrates, and benzenoids were 7.14% (Figure 3A). Twelve differential metabolites were increased, and two were decreased in the serum of the HE group according to heat maps (Figure 3B). These 14 metabolites with significant differences were sorted by log2 (FC); the top-ranked metabolites were all BAs (Figure 3C left). The total BA level in the RP group was significantly lower than that in the HE group (*p* < 0.001) (Figure 3D). A total of 24 different BA types were screened between the HE group and the RP group, among which eight BA types showed significant differences (VIP > 1, *p* < 0.05); the relative abundance of 7-ketolithocholic acid (7-KLCA), cholic acid (CA), chenodeoxycholic acid (CDCA), taurocholic acid (TCA), glycine ursodeoxycholic acid (GUDCA), 12-ketolithocholic acid (12-KLCA), and glycylcholic acid (GCA) in the RP group of cows was significantly lower in the RP than in the HE group (*p* < 0.05), while glycylcholic acid-3 sulfate (glycylcholic acid-3 sulfate) was significantly higher than that in the HE group (*p* < 0.05) (Figure 3E). Among them, 12-KLCA, 7-KLCA, and glycine-3 sulfate showed the highest warning potential according to ROC curve analysis, which can effectively distinguish the HE group from the RP group. Therefore, 12-KLCA, 7-KLCA, and glycine-3 sulfate were selected as potential warning markers for dairy cows with RP (Figure 3C right). 

### 3.5. The KEGG Enrichment Anglyses and Correlation Analyses

**Figure 4:**
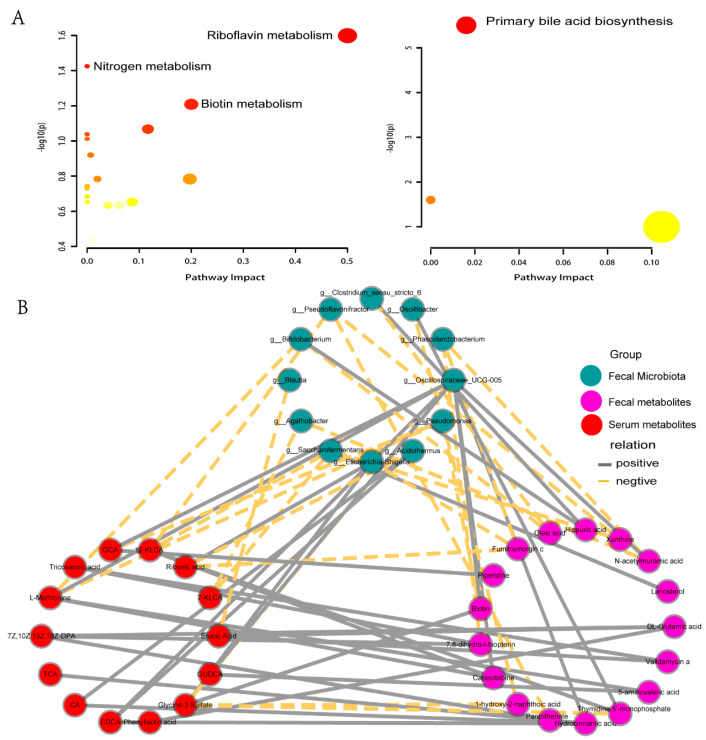
Analysis of metabolic pathways and correlation networks. (**A**) Analysis of metabolic pathways in feces (left) and plasma (right) of cows between the HE and RP groups. The circles represent the different metabolic pathways. The darker circles indicate significant changes for specific metabolites in the corresponding pathway, whereas the size of the circle corresponds to the pathway impact score. (**B**) Correlation analysis between HE group and RP group cows among fecal microbiota, fecal differential metabolites, and serum differential metabolites.

Kyoto Encyclopedia of Genes and Genomes (KEGG) metabolic pathway enrichment analysis of differential metabolites was performed using the MetaboAnalyst 5.0 analysis platform. Eighteen metabolic pathways were enriched in differential metabolites in feces, with significant changes in the riboflavin metabolism and nitrogen metabolic pathways (Figure 4A, left) (*p* < 0.05). The 14 differential metabolites screened in plasma were enriched three metabolic pathways, mainly involving primary BA biosynthesis (Figure 4A, right) (*p* < 0.05). 

The relationship among fecal microbiota, fecal metabolites, and serum metabolites was studied 7 days before calving in dairy cows in the HE and RP groups. Correlation network analysis was used to test the relationship between different metabolites and bacterial genera from different sources (Figure 4B). There was a strong correlation among fecal differential bacteria, fecal differential metabolites, and plasma differential metabolites. Most differences in plasma BAs were negatively correlated with differential metabolites in feces and positively with most fecal differential bacterial genera. Most differential bacterial genera in feces were negatively correlated with most differential metabolites in feces. We focused on analyzing the correlation between potential warning markers. *Oscillospiraceae_UCG-005* was positively correlated with *N*-acetylmuramic (r = 0.58, *p* = 0.008) and GCA (r = 0.51, *p* = 0.02) and negatively with glycine-3-sulfate (r = −0.65, *p* = 0.002). *Escherichia-Shigella* was positively correlated with glycine-3-sulfate (r = 0.58, *p* = 0.008).

## 4. Discussion

At the genus level, two of the best differential genera (*Escherichia-Shigella* and *Oscillospiraceae_UCG-005*) were screened as potential early-warning biomarkers for RP in dairy cows. *Escherichia-Shigella* abundance was significantly higher in the RP group than in the HE group. It belongs to the phylum Proteobacteria and causes multiple infections [20]. It is a Gram-negative bacterium that causes mastitis in dairy cows [21]. There is an abnormal increase in the abundance of *Escherichia-Shigella* in the intestines of obese patients [22]. This may be related to the excessive mobilization of defense systems in dairy cows and the combination of multiple BAs. Increasing evidence shows that endotoxins produced by Gram-negative bacteria are a potential cause of various diseases (including RP) in dairy cows [23]. In contrast, the abundance of *Oscillospiraceae_UCG-005* was significantly higher in the HE group than in the RP group. *Oscillospiraceae_UCG-005* is a microbe that produces short-chain fatty acids (SCFAs), including acetic acid, propionate, and butyrate [24,25], wherein acetic acid reduces liver injury caused by an inflammatory response by inhibiting the TLR4 signaling pathway [26], and propionic acid reduces the LPS-induced inflammatory response and inhibits the activation of TLR-4 protein on the surface of alveolar macrophages [27]. Butyrate can inhibit systemic inflammation induced by high fat intake and provide energy to the intestinal epithelial cells [28]. Therefore, the high levels of *Oscillospiraceae_UCG-005* in the intestinal tract of the HE group can provide energy and reduce the inflammatory response induced by harmful bacteria by producing metabolites, such as SCFAs. In summary, these results indicate that changes in the composition and abundance of the gut microbiota and the expression of metabolites in the HE and RP groups may cause anti-inflammatory and pro-inflammatory reactions. However, there is a lack of verification of inflammation-related indicators. In the early stage of the perinatal period, cows with RP experience a negative energy balance owing to the decrease in dry matter intake (DMI), rumination frequency, and lying time. During this period, they exhibit abnormal glucose and lipid metabolism, and the endogenous microorganisms in the host intestinal tract also change, resulting in an increase in the abundance of pathogenic bacteria and bacterial translocation to the whole body. This results in a chronic inflammatory response [29] that may lead to chorion congestion and edema and increase the incidence of RP.

Numerous studies confirmed that fecal metabolites can reflect the degree of metabolic function of the gut microbiota and their relationship with the host metabolism [30]. We further analyzed and compared the differences in fecal metabolites between the HE and RP groups at 7 days before calving. The differential metabolites were mainly lipids and lipid-like molecules (22.97%). We focused on the differences in host, microbiota, and co-metabolites between the HE and RP groups. *N*-acetylmuramic acid was screened as a potential early-warning marker of RP in dairy cows by a random forest model and ROC curve. *N*-acetylmuramic acid is involved in the formation of a complex polymer network in the peptidoglycan skeleton of bacterial cell walls. Many pathogens use *N*-acetylmuramic acid acetylation and deacetylation of their own cell wall peptidoglycans to escape recognition by the innate immune system [31]. Furthermore, naturally occurring small molecules produced by *N*-acetylmuramic acid-containing peptidoglycans can influence the innate immune response [32,33]. The *N*-acetylmuramic acid concentration in the HE group was significantly higher than that in the RP group. 16S rRNA analysis suggested that this may be related to the significant increase in the abundance of *Escherichia-Shigella* and the significant decrease in the abundance of Gram-positive bacteria (*Oscillospiraceae UCG-005*) in the feces of cows in the RP group. Correlation analysis of differential bacterial genera and metabolites in feces showed that *N*-acetylmuramic acid was strongly positively correlated with *Oscillospiraceae UCG-005* and strongly negatively correlated with *Escherichia-Shigella*, further confirming our hypothesis. Two metabolic pathways (riboflavin and nitrogen metabolism) enriched in fecal differential metabolites showed significant changes in fecal metabolism (*p* < 0.05). Riboflavin (also known as vitamin B2) is an oxidoreductase coenzyme involved in the metabolism of fats, carbohydrates, and proteins. It reduces free radical production and improves oxidative stress [34]. A lack of riboflavin is closely related to endocrine abnormalities (such as those in parathyroid hormone), and the regulation of calcium by parathyroid hormone is particularly important [35]. The high level of riboflavin in the stool of the RP group may be caused by intestinal reabsorption disorder. Nitrogen metabolism is one of the most important biochemical processes in the body, which involves protein synthesis, breakdown, and transport. L-glutamate is involved in nitrogen metabolism. The L-glutamine cycle is the mechanism whereby the liver converts amino acids and tricarboxylic-acid-cycle metabolites. The L-glutamine cycle maintains the nitrogen balance in the body and provides energy [36]. The imbalance of nitrogen metabolism in dairy cows may lead to imbalances in osmotic pressure, disturbance of immune function, and hyperemia and edema of placental tissue [11].

A total of 14 metabolites were identified in plasma. The metabolites with significant differences were mainly lipid-related metabolites, such as BAs (57.14%) and fatty acids (21.48%). Interestingly, lipid metabolites also accounted for the highest proportion of differential fecal metabolites. Our previous studies found that the proportion of lipid metabolites was the highest in cows with abomasum translocation [14] and in pre-ketosis cows with significant differences in plasma and feces metabolites [15]. These results suggest that abnormalities in lipid metabolism in the perinatal period may be related to the development of RP in dairy cows. BAs ranked the highest according to the LOG2FC ranking analysis. We further analyzed the differences in the composition and abundance of plasma BAs between the HE and RP groups. The total BA concentration in the RP group was significantly lower than that in the HE group. Rodents and humans change the size and composition of the total BA pool when suffering from diseases, such as type 2 diabetes [37,38]. Cows with severe hyperketosis show reduced synthesis and secretion of total BAs [39]. Our research findings support the possibility of reduced synthesis and secretion of BAs in RP cows. Primary BAs (CDCA TCA, GCA, and CA) and secondary BAs (7-KLCA, 12-KLCA, and GUDCA) were significantly lower in the RP group than in the HE group, which may be attributed to changes in the composition and function of the gut microbiota. Primary BAs produce secondary BAs under the action of gut microbiota. The 16S rRNA amplicon sequencing also confirmed this hypothesis. Secondary BAs can inhibit the expression of key factors and chemokines involved in inflammatory responses, such as inhibition of NF-kB-mediated dendritic cell activation through TGR5-cAMP-PKA signaling, exhibiting broad anti-inflammatory effects [40,41,42]. Primary BA biosynthesis is a major metabolic pathway in plasma, and the contents of four metabolites involved in this pathway (CDCA, TCA, GCA, and CA) in the HE group were significantly higher than those in the RP group. Primary BAs have positive effects on digestion and absorption of fats, promoting the absorption of fat-soluble vitamins, and regulation of cholesterol and intestinal flora metabolism [43]. The low levels of primary BAs and secondary BAs in the RP group affected the process of lipid absorption. 7-KLCA, 12-KLCA, and glycine-3-sulfate could be used as potential warning markers of RP in dairy cows. In the future, nutrition intervention can be used to adjust the metabolic differences of dairy cows, which has a positive effect on reducing the incidence of RP; however, large-scale follow-up experimental assays are required to address the limited sample size of this study. 

## 5. Conclusions

16S rRNA amplicon sequencing and metabolomics analyses were used in this study. The relative abundance of pathogenic bacteria in the gut of dairy cows in the RP group was increased. Lipids accounted for the highest proportion of differential metabolites in feces and plasma, suggesting that abnormal lipid metabolism may lead to an increased risk of RP in dairy cows. Finally, six types of metabolites were identified from feces and plasma as early-warning markers of RP in dairy cows. These results provide a new strategy to improve the early-warning system and nutritional intervention for RP in dairy cows.

## Data Availability

The 16S rRNA sequencing data have been deposited in the NCBI Sequence Read Archive database (ID: PRJNA1032648 and PRJNA1083747).

## Supplementary Materials

The following supporting information can be downloaded at https://www.mdpi.com/article/10.3390/metabo14070386/s1, Figure S1: Venn diagram of ASV between the healthy and retained placenta groups; Figure S2: Rarefaction curves of fecal microbiota in the healthy and retained placenta groups; Figure S3. Multivariate quality control chart of the fecal untargeted metabolomics; Figure S4. OPLS-DA score plot of the fecal untargeted metabolomics; Figure S5. Permutation test plots of the fecal untargeted metabolomics; Figure S6. Multivariate quality control chart of the serum metabolomics; Figure S7. The orthogonal partial least squares discriminant analysis score chart (left) and permutation test chart (right) of serum metabolomics; Table S1 The feed formula and nutritional components of dairy cows during perinatal period; Table S2: The difference in the relative abundances of fecal microbiota at the phylum level; Table S3: The difference in the abundance of fecal bacterial genera between the HE and RP groups; Table S4: Differential fecal metabolites identified in the HE group vs. RP group in positive and negative mode mass spectrometry. Table S5: Random forest Gini indices and AUC values of the top 15 differential fecal metabolites between the HE and RP groups.

## Abbreviations

BMMC, 1,3-benzenediol, 5-methyl-4-[(1r,6r)-3-methyl-6-(1-methylethenyl)-2-cyclohexen-1-yl]-; BA, bile acid; CA, cholic acid; CDCA, chenodeoxycholic acid; GCA, glycylcholic acid; GUDCA, glycine ursodeoxycholic acid; 7-KLCA, 7-ketolithocholic acid; 12-KLCA, 12-ketolithocholic acid; MHIT, Methanone, [1-(2-hydroxypentyl)-1h-indol-3-yl](2,2,3,3-tetramethylcyclopropyl)-; DDT, 5-(3,4-dihydroxyphenyl)-6,7-dimethyl-5,6,7,8-tetrahydronaphthalene-2,3-diol; MHPN, Methanone, (6-hydroxy-1-pentyl-1h-indol-3-yl)-1-naphthalenyl-; SLGPG, 1-stearoyl-2-linoleoyl-sn-glycero-3-phospho-(1′-rac-glycerol); PDMP, 1-phenyl-2-decanoylamino-3-morpholino-1-propanol; RP, retained placenta; TCA, taurocholic acid.
