# Supplementary material for: Alteration of Fecal Microbiota, Fecal Metabolites, and Serum Metabolites in Dairy Cows with Pre-Retained Placenta
Source: Metabolites. 2024 Jul 15;14(7):386. doi: 10.3390/metabo14070386 (PMC11279091; doi:10.3390/metabo14070386)
Supplement: Supplementary file 1 [file metabolites-14-00386-s001.zip › metabolites-3098225-SI.pdf]

metabolites

MDPI

Supplementary Materials

Alteration of Fecal Microbiota, Fecal Metabolites, and Serum Metabolites in Dairy Cows with Pre-Retained Placenta

Tao Zhou [1,2,†], Zhenlong Du [1,2,†], Zhengzhong Luo [1], Xiaoping Li [3], Dan Wu [1], Yixin Huang [1], Kang Yong [4], Xueping Yao [1], Liuhong Shen [1], Shumin Yu [1], Zuoting Yan [2,*] and Suizhong Cao [1,*]

[1] Department of Clinical Veterinary Medicine, College of Veterinary Medicine, Sichuan Agricultural University, Chengdu 611130, China
[2] Lanzhou Institute of Animal Husbandry and Veterinary Pharmaceutical, Chinese Academy of Agricultural Sciences, Lanzhou 730050, China
[3] Department of Clinical Veterinary Medicine, College of Veterinary Medicine, China Agricultural University, Beijing 100000, China
[4] Department of Animal Husbandry & Veterinary Medicine, College of Animal Science and Technology, Chongqing Three Gorges Vocational College, Chongqing 404105, China
* Correspondence: yanzuoting@caas.cn (Z.Y.); suizhongcao@sicau.edu.cn (S.C.)
† These authors contributed equally to this work.

Citation: Zhou, T.; Du, Z.; Luo, Z.; Li, X.; Wu, D.; Huang, Y.; Yong, K.; Yao, X.; Shen, L.; Yu, S.; et al. Alteration of Fecal Microbiota, Fecal Metabolites, and Serum Metabolites in Dairy Cows with Pre-Retained Placenta. *Metabolites* **2024**, *14*, 386. https://doi.org/10.3390/metabo14070386

Academic Editor: Diming Wang

Received: 25 June 2024
Revised: 11 July 2024
Accepted: 12 July 2024
Published: 15 July 2024

Copyright: © 2024 by the authors. Licensee MDPI, Basel, Switzerland. This article is an open access article distributed under the terms and conditions of the Creative Commons Attribution (CC BY) license (https://creativecommons.org/licenses/by/4.0/).

1. Supplementary Methods

1.1. Fecal untargeted metabolomic analysis

LC/MS conditions: The supernatant was subjected to ultra-high-performance liquid chromatography (1290 Infinity II; Agilent Technologies, Santa Clara, CA, USA). The column temperature was 25°C and the flow rate was 0.3 mL/min. The injection volume was 2 μL. The mobile phase consisted of A (water, 25 mM ammonium acetate, and 25 mM ammonia) and B (acetonitrile). The gradient elution procedure was as follows: 0 to 1.5 min, 98 % B (acetonitrile); 1.5 to 12 min, B from 98 % linear change to 2 %; 12 to 14 min, B maintained at 2 %; 14 to 14.1 min, B from 2 % linear change to 98 %; 14.1 to 17 min, B maintained at 98 %. The autosampler was maintained at 4 °C throughout the reaction. During the entire analysis process, a random sequence was adopted, and QC samples were inserted into the analysis process to monitor and evaluate the stability of the system and the reliability of the experimental data.

ESI-MS/MS conditions: A Q Exactive series mass spectrometer was used for mass spectrometry analysis. The spray voltage and ion source temperature were set to 600 °C and 5500 V in positive and negative ion modes, respectively; the first-level mass-to-charge ratio detection range was 80–1,200 Da, resolution was 60,000, scanning cumulative time was 100 ms, and the second-level adopted a segmented acquisition method with a scanning range of 70–1,200 Da, secondary resolution of 30,000, and scan accumulation time of 50 ms.

1.2. Serum metabolomic analysis

Chromatographic conditions: The samples were separated using Agilent 1290 Infinity LC ultra-high performance liquid chromatography (UHPLC) HILIC and C18 columns; HILIC chromatography column temperature 35 ℃; Flow rate of 0.3mL/min; Injection volume 2 μ L; Mobile phase composition A: 90% water+2mM ammonium formate+10% acetonitrile, B: methanol+0.4% formic acid; The gradient elution procedure is as follows: 0-1.0 min, 85% B; From 1.0 to 3.0 minutes, B changes linearly from 85% to 80%; 3.0-4.0 minutes, 80% B; 4.0-6.0 minutes, B changes linearly from 80% to 70%; 6.0-10.0 minutes, B changes linearly from 70% to 50%; 10-15.5 minutes, B maintained at 50%; From 15.5 to 15.6 minutes, B changes linearly from 50% to 85%; From 15.6 to 23 minutes, B remained at 85%. C18 chromatography column temperature: 40 ℃; Flow rate of 0.4mL/min; Injection volume 2 μ L; Mobile phase composition A: water+5 mM ammonium acetate+0.2% ammonia

water, B: 99.5% acetonitrile+0.5% ammonia water; The gradient elution procedure is as follows: 0-5 minutes, B changes linearly from 5% to 60%; 5-11 minutes, B changes linearly from 60% to 100%; 11-13 minutes, B maintained at 100%; 13-13.1 minutes, B changes linearly from 100% to 5%; 13.1-16 minutes, B maintained at 5%; During the entire analysis process, the sample was placed in a 4 ℃ automatic sampler. To avoid the impact of signal fluctuations in instrument detection, continuous analysis of samples is carried out in random order. Insert QC samples into the sample queue for monitoring and evaluating the stability of the system and the reliability of experimental data.

Mass spectrometry conditions: Mass spectrometry analysis was performed using the AB 6500 QTRAP mass spectrometer (AB SCIEX). The ESI source conditions are as follows: Source temperature: 580 ℃, Ion Source Gas1 (GS1): 45, Ion Source Gas2 (GS2): 60, Current Gas (CUR): 35, Ion Spray Voltage (IS):+4500 V or -4500 V in positive or negative modes, observed, and monitored using MRM mode.

2. Supplementary Figures and Tables

2.1. Supplementary Figures

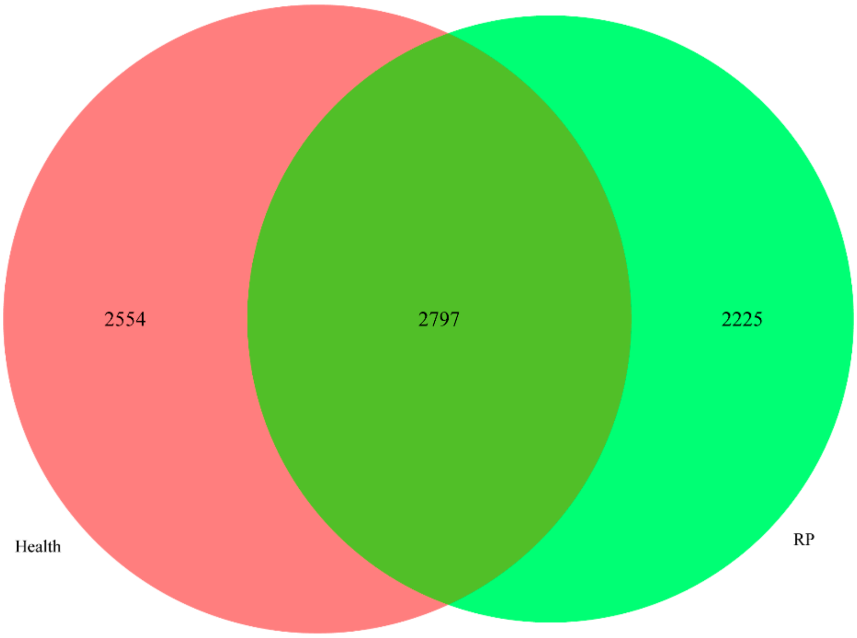

Figure S1. The Venn diagram of ASV between the health and Retained placenta group.

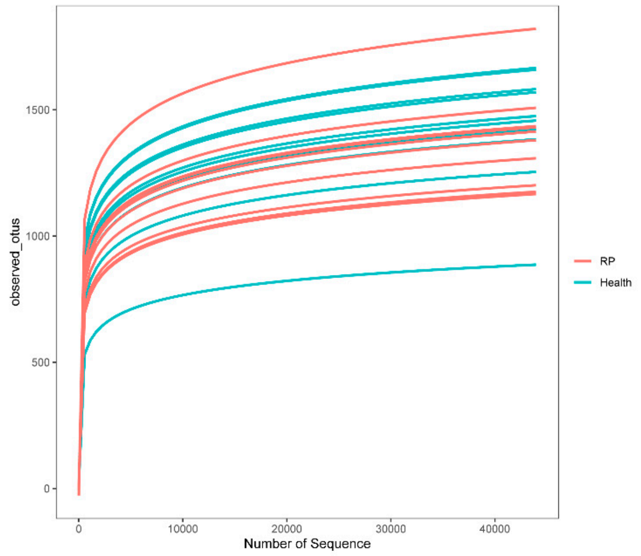

Figure S2. The rarefaction curves of fecal microbiota health and retained placenta group.

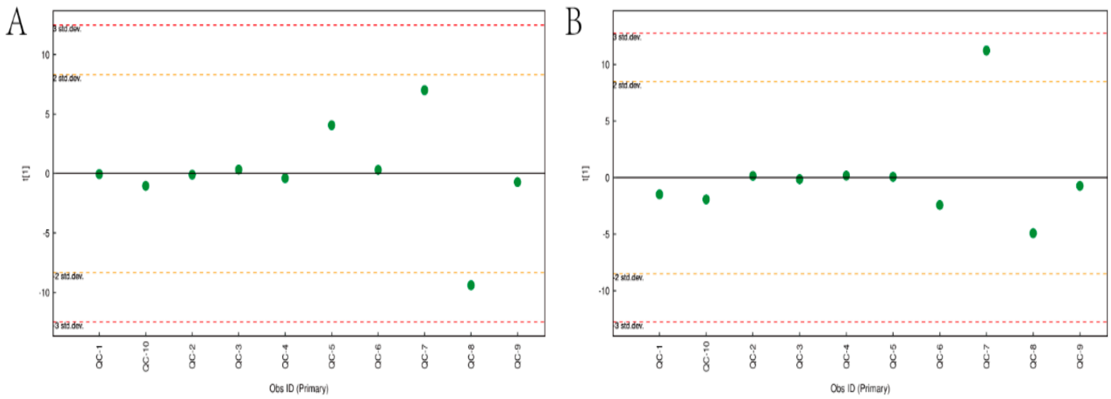

Figure S3. Multivariate quality control chart of the fecal untargeted metabolomics

Note: A and B are multivariate quality control charts (n=10) in positive-negative ion mode, respectively. Green dots indicate individual samples, and orange and red dashed lines indicate biological quality control scope limits

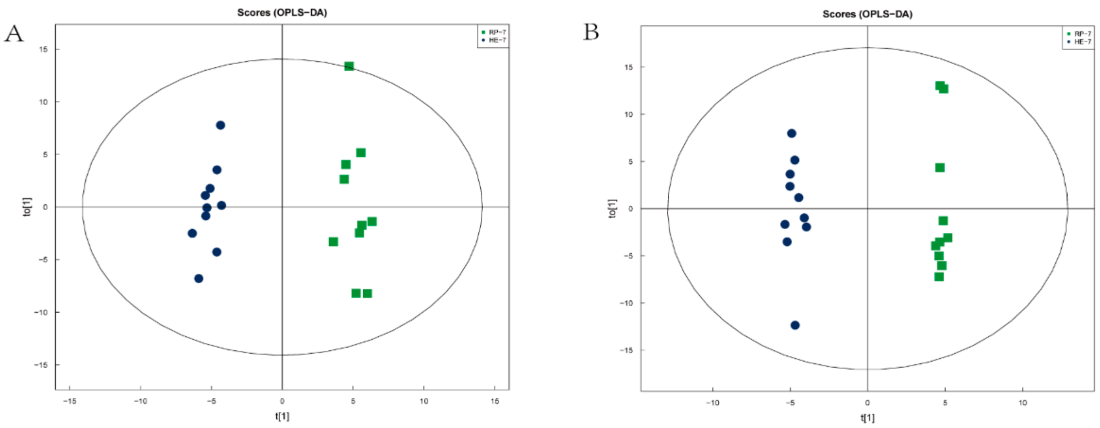

Figure S4. OPLS-DA score plot of the fecal untargeted metabolomics

Note: A and B are the OPLS-DA scores of the HE group (n=10) and the RP group (n=10) in positive and negative ion mode, respectively.

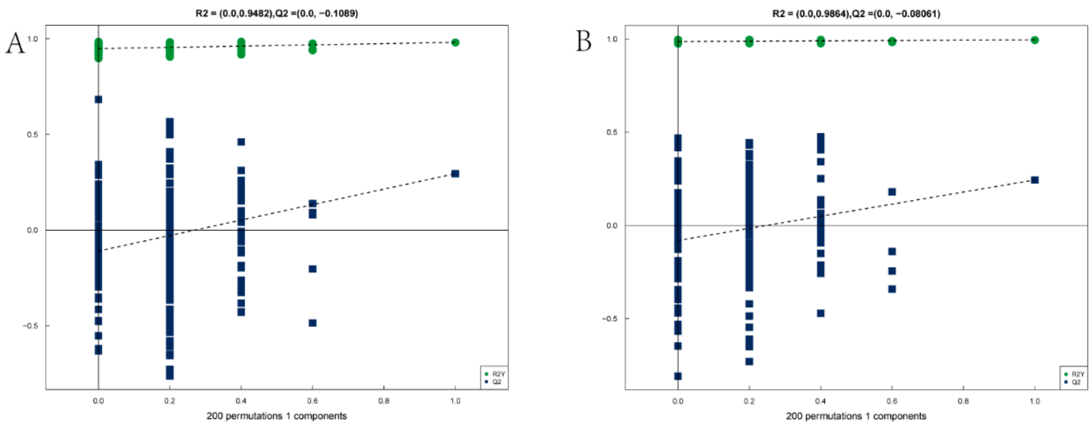

Figure S5. Permutation test plots of the fecal untargeted metabolomics

Note: A is the permutation test diagram of the overall samples in positive ion mode; B is the permutation test diagram of the overall samples in negative ion mode.

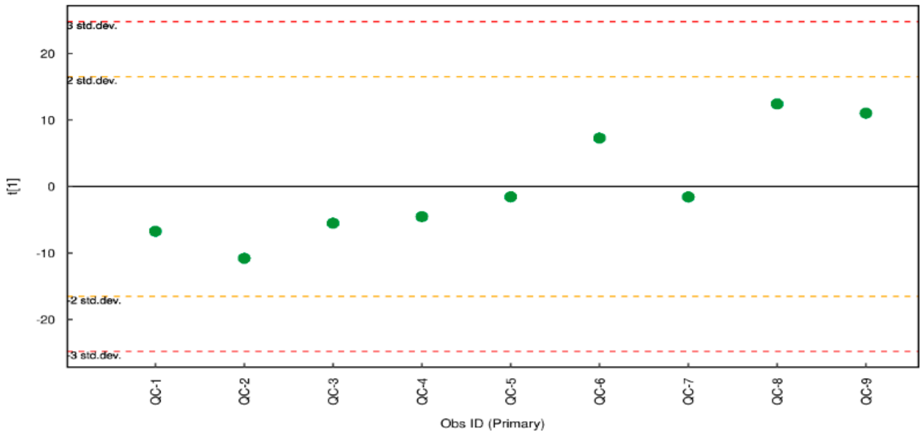

Figure S6. Multivariate quality control chart of the serum metabolomics

Note: Green dots represent individual samples (n=10), and orange and red dotted lines represent the limits of biological quality control range.

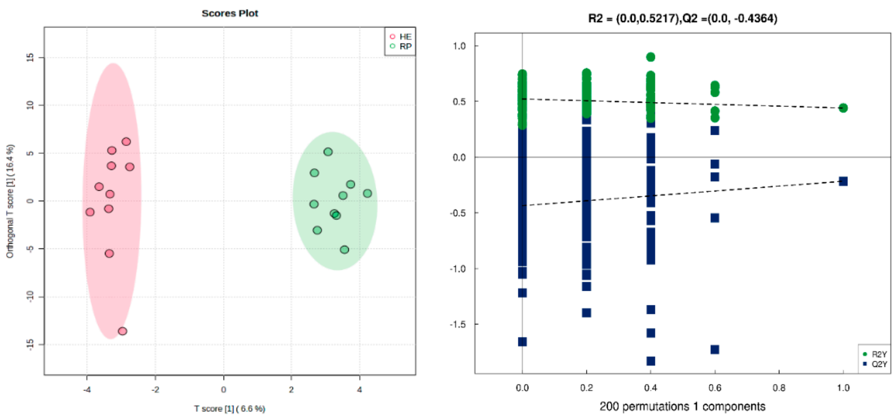

Figure S7. The orthogonal partial least squares discriminant analysis score chart (left) and permutation test chart (right) of serum metabolomics

Note: The left panel shows the orthogonal partial least squares discriminant analysis (OPLS-DA) of plasma samples. The pink and green dots represent HE (n=10) and RP samples (n=10), respectively. The two groups of samples are significantly separated without any overlap, indicating the successful construction of the model in the study. The right panel shows the predictive ability of the model. In order to avoid overfitting of the model, 999 random permutation tests were conducted to cross validate the intercepts of feces and plasma from these three components, thereby proving the effectiveness of the model.

2.2. Supplementary Tables

Table S1. The feed formula and nutritional components of dairy cows during perinatal period.

Item	Prepartum	Postpartum
% of DM		
corn silage	29.04	18.80
oat hay	32.67	4.32
alfalfa hay	-	17.27
wheat straw	7.26	-
choline	0.44	-
complete feeds[1]	27.29	-
complete feeds (Lactation)[2]	-	32.61
anionic salt	1.85	-
steam flaked corn	-	12.95
soybean meal	1.45	-
cottonseed	-	6.47
beet pulp	-	6.48
molasses	-	1.10
total	100	100
% of DM unless noted		
DM	50.23	50.63
CP	15.3	18.5
NDF	45.71	39.27
ADF	30.4	28
Ca	0.57	0.98
P	0.4	0.53
NE_L, Mcal/kg of DM	1.41	1.74

Note: Guaranteed value (%) of composition of each kilogram of premix: crude protein≥23.5, crude fat≥2.0, crude fiber≤9.0, calcium 0.75-1.5, total phosphorus ≥0.3, lysine≥ 0.6, sodium chloride 0.1-1.5; 2 Guaranteed value (%) of composition analysis for each kilogram of premix: crude protein≥19.0, crude fiber≤13.0, calcium 0.70-1.80, total phosphorus≥0.5, lysine≥ 0.6, sodium chloride 0.3-1.5; The net energy of lactation is the calculated value, and others are the measured value. 1. Anionic salt information: animate (Alibao) is provided by International Mineral Services (IMS) in the United States, with measured values of sulfur 7.5%, chlorine 16.2%, and magnesium 6.6%. The DCAD anion cation difference is calculated based on the formula (% Na/0.023)+(% K/0.039) - (% Cl/0.0355) - (% S/0.016). (DCAD level: -126.24mEq/kg. DM).

Table S2. The difference in the relative abundances of fecal microbiota at phylum level.

NO.	Phylum	Groups (n = 10)		SEM	*P*-value
		Health	RP		
1	Firmicutes	72.90	72.37	0.505	0.71
2	Bacteroidota	21.09	21.32	0.622	0.82
3	Verrucomicrobiota	1.93	1.89	0.218	0.94
4	Proteobacteria	1.11	1.70	0.162	0.05
5	Spirochaetota	0.48	0.62	0.063	0.36
6	Actinobacteriota	0.36	0.36	0.043	0.82
7	Bacteroidetes	0.50	0.39	0.032	0.11
8	Patescibacteria	0.40	0.37	0.043	0.94
9	Fusobacteriota	0.05	0.08	0.014	0.29
10	Cyanobacteria	0.28	0.24	0.037	0.71
11	Desulfobacterota	0.21	0.11	0.031	0.06
12	unclassified	0.21	0.21	0.031	/
13	Acidobacteriota	0.17	0.10	0.012	＜0.001
14	Planctomycetota	0.06	0.06	0.012	0.62
15	Fibrobacterota	0.08	0.06	0.013	0.60
16	Actinobacteria	0.03	0.06	0.011	0.23
17	Campylobacterota	0.03	0.02	0.004	0.03
18	Acidobacteria	0.04	0.02	0.004	0.21
19	Chloroflexi	0.02	0.01	0.003	0.29
20	WPS-2	0.03	0.00	0.004	＜0.001
21	Elusimicrobiota	0.01	0.00	0.505	0.47

Table S3. The difference in the abundance of fecal bacterial genus taxa between HE group and RP group.

NO.	geuns	Relative abundance＞0.05 %		SEM	P-value
		HE	RP		
1	*Oscillospiraceae_UCG-005*	20.81	18.36	0.36	＜0.05
2	*Phascolarctobacterium*	0.67	0.94	0.05	0.03
3	*Pseudoflavonifractor*	0.69	0.88	0.04	0.02
4	*Agathobacter*	0.48	0.76	0.06	0.02
5	*Acidothermus*	0.03	0.07	0.01	＜0.05
6	*Oscillibacter*	0.54	0.44	0.02	0.03
7	*Saccharofermentans*	0.18	0.33	0.03	0.02
8	*Escherichia-Shigella*	0.06	0.41	0.05	＜0.05
9	*Veillonella*	0.14	0.11	0.01	0.03
10	*Pseudomonas*	0.003	0.18	0.06	0.03
11	*Bifidobacterium*	0.07	0.11	0.04	＜0.05
12	*Blautia*	0.09	0.06	0.01	0.05
13	*Clostridium_sensu_stricto_6*	0.01	0.11	0.04	0.01

Table S4. Differential fecal metabolites identified of HE group vs. RP group in the positive and negative mode.

No.	Metabolite	VIP	Fold change	p-value	HMDB	KEGG
1	Nootkatone	3.766066	0.577285	0.001376		C17914
2	Cis-hydroxyperhexiline	2.21553	0.694506	0.005829		
3	1,3-benzenediol, 5-methyl-4-[(1r,6r)-3-methyl-6-(1-methylethenyl)-2-cyclohexen-1-yl]-	1.205115	0.821664	0.006676		
4	Pyrrolidine	3.939947	0.812699	0.007306	HMDB0031641	
5	4-aminobiphenyl	1.238442	0.789942	0.007816	HMDB0013195	C10998
6	Uncarine c	1.072302	2.003187	0.008452		C17595
7	Pantothenate	1.30438	0.644372	0.010901	HMDB0000210	C00864
8	Imazamox	2.028584	0.680086	0.010961		C18598
9	N-.alpha.-(tert-butoxycarbonyl)-l-valine	1.875114	0.627679	0.012043		
10	D-mannosamine	9.840382	1.724506	0.012145		C03570
11	Propentofylline	1.302408	0.754124	0.013421	HMDB0014645	C01814
12	Simeconazole	1.543457	0.761298	0.013544		C18544
13	Leucylleucine	2.212225	1.411956	0.014803	HMDB0028933	C11332
14	7,8-dihydro-l-biopterin	1.145339	0.641387	0.017722	HMDB0000038	C02953
15	Fenfluramine	4.047611	2.003541	0.019098		C06996
16	Fumitremorgin c	1.910932	0.529949	0.019813	HMDB0038642	C20604
17	Cinchonine	2.794276	0.804279	0.024194	HMDB0030282	C06528
18	(cis+trans)-nerodilol	1.25092	0.88613	0.024485		C09704
19	Flusilazole	3.031616	0.70713	0.025979	HMDB0039815	C18733
20	Methanone, [1-(2-hydroxypentyl)-1h-indol-3-yl](2,2,3,3-tetramethylcyclopropyl)-	1.22033	0.594471	0.026883		
21	2-(2',3',4'-trihydroxybutyl) quinoxaline	1.359847	0.793778	0.027312		
22	Estra-1,3,5(10),7-tetraene-3,17. beta. - diol	3.144564	0.771179	0.028801		C14485
23	Neolinustatin	1.477705	0.739225	0.030094		C08336
24	Capecitabine	1.845947	0.83064	0.033079	HMDB0015233	C12650
25	Vincanidine	1.514769	0.763011	0.033189		
26	Vitexin	1.25533	2.376507	0.033455		C01460
27	Fluvastatin	3.247573	1.722257	0.033965		C07014
28	Oxycodone	2.305289	0.775239	0.035938	HMDB0014640	C08018
29	Narirutin	1.074988	0.676957	0.036212		C09793
30	Piperidine	2.59819	0.699334	0.036646	HMDB0034301	C01746
31	Thymidine 5'-monophosphate	1.049625	0.686779	0.037746	HMDB0001227	C00364
32	1-stearoyl-2-linoleoyl-sn-glycero-3-phospho-(1'-rac-glycerol)	2.068559	1.666116	0.038285		
33	2-[methyl(4-piperidinyl) amino] ethanol	1.292567	0.620346	0.039127		
34	Leu-Phe	1.712455	1.413677	0.040308		
35	1-phenyl-2-decanoylamino-3-morpholino-1-propanol	4.987641	0.264183	0.040413		
36	Irbesartan	2.112061	1.437627	0.042909	HMDB0015163	C07469
37	DL-Glutamic acid	1.91808	0.80635	0.044382	HMDB0060475	C00025
38	Validamycin a	1.593346	0.644413	0.045505		C12112
39	Quinine	2.771284	0.653621	0.045883	HMDB0014611	C06526
40	(-)-riboflavin	2.58136	1.75155	0.045883	HMDB0000244	C00255

41	5-aminovaleric acid	3.94154	0.872332	0.046714	HMDB0003355	C00431
42	Indole	3.507931	0.629792	0.047158	HMDB0000738	C00463
43	Palmitoleoyl 3-carba-cyclic phosphatidic acid	1.606204	2.294478	0.049168		
44	17alpha-ethynylestradiol	2.513105	0.755158	0.049238	HMDB0001926	C07534
45	Purine	8.109929	0.62673	0.002964	HMDB0001366	C15587
46	N-acetylmuramic acid	1.450583	0.644513	0.003861	HMDB0060493	C02713
47	Gln-glu	1.250916	0.705932	0.005717		
48	Nonanoic acid	4.052596	0.80319	0.008191	HMDB0000847	C01601
49	2'-o-methyladenosine	3.254189	2.069366	0.008669	HMDB0004326	C04779
50	Trans-3'-hydroxycotinine o-.beta.-d-glucuronide	2.184374	0.438286	0.008766		
51	Azelaic acid	3.108591	0.75105	0.009988	HMDB0000784	C08261
52	Physcion	1.330692	0.508055	0.013219		C17045
53	5-(3,4-dihydroxyphenyl)-6,7-dimethyl-5,6,7,8-tetrahydronaphthalene-2,3-diol	1.285908	0.472238	0.018062		
54	4,6-dinitro-o-cresol	3.093274	2.838358	0.018119		C18653
55	Hippuric acid	5.230062	1.69299	0.019436	HMDB0000714	C01586
56	Lanosterol	1.58026	1.569484	0.019901	HMDB0001251	C01724
57	Podocarpic acid	7.705606	1.702287	0.020659		C09171
58	(-)-hydroxycitric acid lactone	1.413348	0.520906	0.021474		
59	Hydrocinnamic acid	13.37482	0.721015	0.022473	HMDB0000764	C05629
60	Methanone, (6-hydroxy-1-pentyl-1h-indol-3-yl)-1-naphthalenyl-	3.331889	0.431712	0.023292		
61	Caproic acid	2.775059	0.765779	0.024146	HMDB0000535	C01585
62	Ile-Leu	1.440386	1.393138	0.025909		
63	N-palmitoyl-d-erythro-dihydroceramide-1-phosphate	4.132652	1.446761	0.02662	HMDB0010698	
64	Picolinic acid	1.75282	0.700933	0.027239	HMDB0002243	C10164
65	1-hydroxy-2-naphthoic acid	2.063972	2.34786	0.027794		C03203
66	Forsythoside e	1.024408	0.521773	0.030017		
67	Glabrolide	6.078118	1.585528	0.030792		
68	Xanthine	4.624257	0.621688	0.030976	HMDB0000292	C00385
69	Ala-Ala	3.735691	0.702943	0.032654	HMDB0000303	C00398
70	Lauric isopropanolamide	2.079099	1.309635	0.032995		
71	Oleic acid	21.69365	0.713697	0.037067	HMDB0000207	C00712
72	Phenylbenzimidazolesulfonic acid	1.063855	2.984435	0.03748		
73	Biotin	2.411821	0.625923	0.042122	HMDB0000030	C00120
74	3.alpha.-hydroxy-7-oxo-5.beta.-cholanic acid	2.438683	2.032995	0.047551		

Table S5. Random forest Gini indices and AUC values of top 15 fecal differential metabolites between the HE and RP groups.

No.	Differential metabolites	Class	AUC	Gini
1	N-acetylmuramic acid	Organic oxygen compounds	0.82	0.93
2	Lanosterol	Lipids and lipid-like molecules	0.80	0.84
3	Hippuric acid	Benzenoids	0.83	0.82
4	Fumitremorgin c	Organoheterocyclic compounds	0.80	0.70
5	7-8-dihydro-l-biopterin	Organoheterocyclic compounds	0.82	0.70
6	Piperidine	/	0.75	0.61
7	Thymidine 5‘-monophosphate	Nucleosides, nucleotides, and analogues	0.78	0.56

8	Riboflavin	Organoheterocyclic compounds	0.74	0.49
9	Pantothenate	Organic oxygen compounds	0.81	0.46
10	5-aminovaleric acid	Organic acids and derivatives	0.75	0.45
11	Biotin	/	0.77	0.44
12	1-hydroxy-2-naphthoic acid	Benzenoids	0.76	0.42
13	Indole	Organoheterocyclic compounds	0.75	0.39
14	Xanthine	Organoheterocyclic compounds	0.77	0.38
15	Validamycin-a	Organic oxygen compounds	0.77	0.35
